# A Phase I study of AD5-GUCY2C-PADRE in stage I and II colon cancer patients

**DOI:** 10.1186/2051-1426-3-S2-P450

**Published:** 2015-11-04

**Authors:** Adam Snook, Trevor Baybutt, Michael Mastrangelo, Nancy Lewis, Scott Goldstein, Walter Kraft, Yaa Oppong, Terry Hyslop, Ronald Myers, Vitali Alexeev, Laurence Eisenlohr, Takami Sato, Scott Waldman

**Affiliations:** 1 Thomas Jefferson University, Philadelphia, PA, USA; 2 Duke University, Durham, NC, USA

## Background

Ad5-GUCY2C-PADRE is a replication-deficient human type 5 recombinant adenovirus (Ad5) vaccine encoding guanylyl cyclase C (GUCY2C) fused to the PAn DR Epitope (PADRE). GUCY2C, a paracrine hormone receptor producing the second messenger cyclic GMP (cGMP), is selectively expressed by intestinal epithelial cells and a subset of hypothalamic neurons, but not other tissues. Importantly, GUCY2C is over-expressed in nearly all primary and metastatic human colorectal tumors. Preclinical studies in mice demonstrated selective tolerance of GUCY2C-specific CD4+ T cells, but not CD8+ T or B cells, necessitating inclusion of the exogenous CD4+ T helper cell epitope PADRE to maximize GUCY2C-specific CD8+ T cell and antibody responses and antitumor efficacy, without autoimmunity.

## Patients and methods

This is an open-label, single arm “proof-of-concept” study evaluating a single dose level of Ad5-GUCY2C-PADRE as a vaccine for surgically-treated, node-negative colon cancer subjects (NCT01972737). Patients received a single intramuscular administration of 10^11^ Ad5-GUCY2C-PADRE viral particles. Safety and immunomonitoring where examined at 30, 90 and 180 days following vaccination. Primary objectives were to determine the safety, tolerability and toxicity of Ad5-GUCY2C-PADRE and to determine whether Ad5-GUCY2C-PADRE induces GUCY2C-specific immune responses. The study employed a joint efficacy-toxicity design and included stopping rules for either efficacy or toxicity. Results here were obtained during the planned interim analysis following accrual of 10 subjects.

## Results

The vaccine was well tolerated, producing only mild adverse events (AEs). Short-lived injection site pain/swelling, body aches and chills were the most commonly observed AEs and occurred in 30-40% of subjects. GUCY2C-specific antibody and T-cell responses were observed in a subset of subjects. Consistent with preclinical mouse data, T-cell responses were composed of CD8+, but not CD4+, T cells. Importantly, GUCY2C-specific responses occurred only in subjects with low Ad5 neutralizing antibody (NAb) titers at the time of vaccination, suggesting that pre-existing Ad5 immunity limits Ad5-GUCY2C-PADRE immunogenicity.

## Conclusions

Interim analysis of 10 subjects receiving Ad5-GUCY2C-PADRE demonstrates proof-of-concept that GUCY2C is immunogenic in humans and that GUCY2C-directed vaccination is safe. Moreover, the presence of GUCY2C-specific antibody and CD8+ T-cell, but not CD4+ T-cell, responses is consistent with selective CD4+ T-cell tolerance observed in mouse models. These data establish GUCY2C as a safe and immunogenic target for immunotherapy in cancer patients.

## Trial registration

ClincialTrials.gov identifier NCT01972737.

